# Genome-scale metabolic model of the versatile bacterium *Paracoccus denitrificans* Pd1222

**DOI:** 10.1128/msystems.01077-23

**Published:** 2024-01-05

**Authors:** Sergio Bordel, Diego Martín-González, Tim Börner, Raúl Muñoz, Fernando Santos-Beneit

**Affiliations:** 1 Department of Chemical Engineering and Environmental Technology, School of Industrial Engineering, University of Valladolid, Valladolid, Spain; ^2^Institute of Sustainable Processes, Valladolid, Spain; 3 HES-SO Valais/Wallis, School of Engineering, Institute of Life Technologies, Sion, Switzerland; University of British Columbia, Vancouver, Canada

**Keywords:** metabolism, *Paracoccus denitrificans*, denitrification, genome scale metabolic models, biodegradation

## Abstract

A genome scale metabolic model of the bacterium *Paracoccus denitrificans* has been constructed. The model containing 972 metabolic genes, 1,371 reactions, and 1,388 unique metabolites has been reconstructed. The model was used to carry out quantitative predictions of biomass yields on 10 different carbon sources under aerobic conditions. Yields on C1 compounds suggest that formate is oxidized by a formate dehydrogenase O, which uses ubiquinone as redox co-factor. The model also predicted the threshold methanol/mannitol uptake ratio, above which ribulose biphosphate carboxylase has to be expressed in order to optimize biomass yields. Biomass yields on acetate, formate, and succinate, when NO_3_^−^ is used as electron acceptor, were also predicted correctly. The model reconstruction revealed the capability of *P. denitrificans* to grow on several non-conventional substrates such as adipic acid, 1,4-butanediol, 1,3-butanediol, and ethylene glycol. The capacity to grow on these substrates was tested experimentally, and the experimental biomass yields on these substrates were accurately predicted by the model.

**IMPORTANCE:**

*Paracoccus denitrificans* has been broadly used as a model denitrifying organism. It grows on a large portfolio of carbon sources, under aerobic and anoxic conditions. These characteristics, together with its amenability to genetic manipulations, make *P. denitrificans* a promising cell factory for industrial biotechnology. This paper presents and validates the first functional genome-scale metabolic model for *P. denitrificans*, which is a key tool to enable *P. denitrificans* as a platform for metabolic engineering and industrial biotechnology. Optimization of the biomass yield led to accurate predictions in a broad scope of substrates.

## INTRODUCTION

*Paracoccus denitrificans* was first isolated from soil, more than one century ago, as *Micrococcus denitrificans* (1). The original selection of this species was based on its ability to convert nitrate into molecular nitrogen. However, *P. denitrificans* was brought to the attention of the scientific community when John and Whatley (2) concluded that this bacterium resembled a mitochondrion more closely than other bacteria. These authors suggested that an evolutionary transition from the plasma membrane of an ancestral bacterium resembling *P. denitrificans* to the inner mitochondrial membrane was feasible. Certainly, *P. denitrificans* effectively assembles in a single organism those features of the mitochondrial respiratory chain and oxidative phosphorylation (i.e., electron transport flavoproteins, NADH-ubiquinone oxidoreductase, aa3-type terminal cytochrome oxidase, bc1 complex, and c-type cytochromes) that are otherwise randomly distributed among most other aerobic bacteria. Indeed, most of these features are shared with other members of the alpha-proteobacteria, but it is clear that *P. denitrificans* is one of the best-known examples (3).

The interest in *P. denitrificans* is not only related to its electron transport chain but also on its metabolic flexibility. *P. denitrificans* is a model denitrifying organism. Denitrification is important for soil fertility, greenhouse gas emission, as well as in waste and water treatment processes. The versatile metabolic capabilities of *P. denitrificans*, as well as its capability to tolerate salt concentrations higher than 3% (4) and to form biofilms on surfaces at the air-liquid interface under static conditions, prompt the use of *P. denitrificans* for bioremediation, especially in water treatment processes, in which microorganisms frequently grow as surface-attached biofilms or as granules (5).

Despite being rather ubiquitous, *P. denitrificans* is found more often in environments that are subject to fluctuating conditions, such as soil, sewage, or sludge (6). In order to survive in such dynamic habitats, *P. denitrificans* cells accumulate storage materials in the form of polyhydroxyalkanoates (PHAs), which, due to their role as carbon and energy storage materials, endow the bacterium with enhanced survival capabilities under adverse environmental conditions (7). PHAs are being exploited by the industry for numerous applications (8). *P. denitrificans* synthesizes poly (3-hydroxybutyric acid) (PHB), poly (3-hydroxyvaleric acid), and the poly (3-hydroxybutyric acid-co-3-hydroxyvaleric acid) copolyester from different carbon sources, including glycerol (9). Glycerol constitutes one of the main by-products obtained during transesterification of vegetable oil to biodiesel, and its disposal is quite expensive and problematic for the environment. *P. denitrificans* can accumulate up to 70% of PHB from glycerol when grown in a 2.5-L fed batch bioreactor (10). Microbial fermentation of glycerol to valuable products, such as PHAs, offers an economic and ecologic solution for its disposal.

Few new isolates of *P. denitrificans* have been found (11) since the first strain was isolated by Beijerinck (1). Most molecular work has been performed on a single strain, *P. denitrificans* Pd1222, a mutant with increased conjugation frequencies and pleiotropic loss of a (nGATCn)-DNA-modifying property, which is more amenable to genetic manipulation than other relative strains (12). The molecular biology of the genus has developed since then, and a considerable amount of new information has become available in the last two decades (13, 14). Actually, *P. denitrificans*, similarly to other bacterial models such as *Escherichia coli* and *Bacillus subtilis*, is an extremely powerful model system because introducing defined genetic changes such as gene fusions, deletions and other genetic manipulations is straightforward and fast (15).

Genome-scale metabolic models (GSMMs) are comprehensive compilations of all metabolic reactions taking place in a particular cell and can be used to predict theoretical yields on different carbon sources, as well as the effects of genetic manipulations, such as the expression of heterologous genes or gene knockouts. The model presented here, together with all previously described metabolic and genetic characteristics, could facilitate the use of *P. denitrificans* as a platform for metabolic engineering and industrial biotechnology.

To our knowledge, there is only one automatically generated GSMM of *P.denitrificans* Pd1222 publicly available, which lacks the capacity of simulating growth in any single carbon source: https://github.com/cdanielmachado/embl_gems/tree/master/models/p/paracoccus.

An initial draft of our model was previously used to simulate the fluxes around the acetyl-CoA node during the lag and exponential growth phases (16), leading to the conclusion that, during the lag phase, *P. denitrificans* dedicates all the acetyl-CoA that it produces for the synthesis of PHB, while during exponential growth, acetyl-CoA is produced at the same rate as in the lag phase, but most of it is used to feed the Tricarboxylic Acids (TCA) cycle.

## RESULTS

### Metabolic model of *Paracoccus denitrificans*

A GSMM of *Paracoccus denitrificans* Pd1222 was constructed by annotating its genome (GenBank GCA_000203895.1) using RAST (17) and building an initial draft model with SEED (18). The draft generated by SEED was manually curated. The resulting model contains 972 metabolic genes, 1,371 reactions (including transport reactions), and 1,388 unique metabolites. The model, in SBML and text formats, together with the genome annotation obtained from RAST, is publicly available at https://github.com/SergioBordel/ModelParacoccus.

### Prediction of aerobic biomass yields in chemostats

The reconstructed GSMM was used to compute the optimal theoretical biomass yields of *Paracoccus denitrificans* on three different carbon sources (mannitol, succinate, and glycerol) under aerobic conditions, using flux balance analysis (FBA). The specific substrate consumption rate (*q_s_*) of a microbial culture follows Pirt’s equation (19).


(1) $$q_{s}=m_{s}+\frac{1}{Y^{max}}\mu$$

where *µ* is the specific growth rate, *m_s_* is the maintenance substrate consumption rate, and *Y*^max^ is the maximal biomass yield (g-DW mol^−1^) or the yield that would be obtained in the absence of non-growth-associated ATP consumption. A theoretical value for this maximal yield can be estimated using FBA, by constraining the substrate consumption to one and optimizing biomass production without ATP maintenance costs. Ammonium was used as nitrogen source for the simulations. The assumption of biomass yield optimization is broadly used to predict metabolic flux distributions by FBA, even if it tends to be accurate only under substrate-limiting conditions, such as those in a chemostat (20).

**Fig 1:**
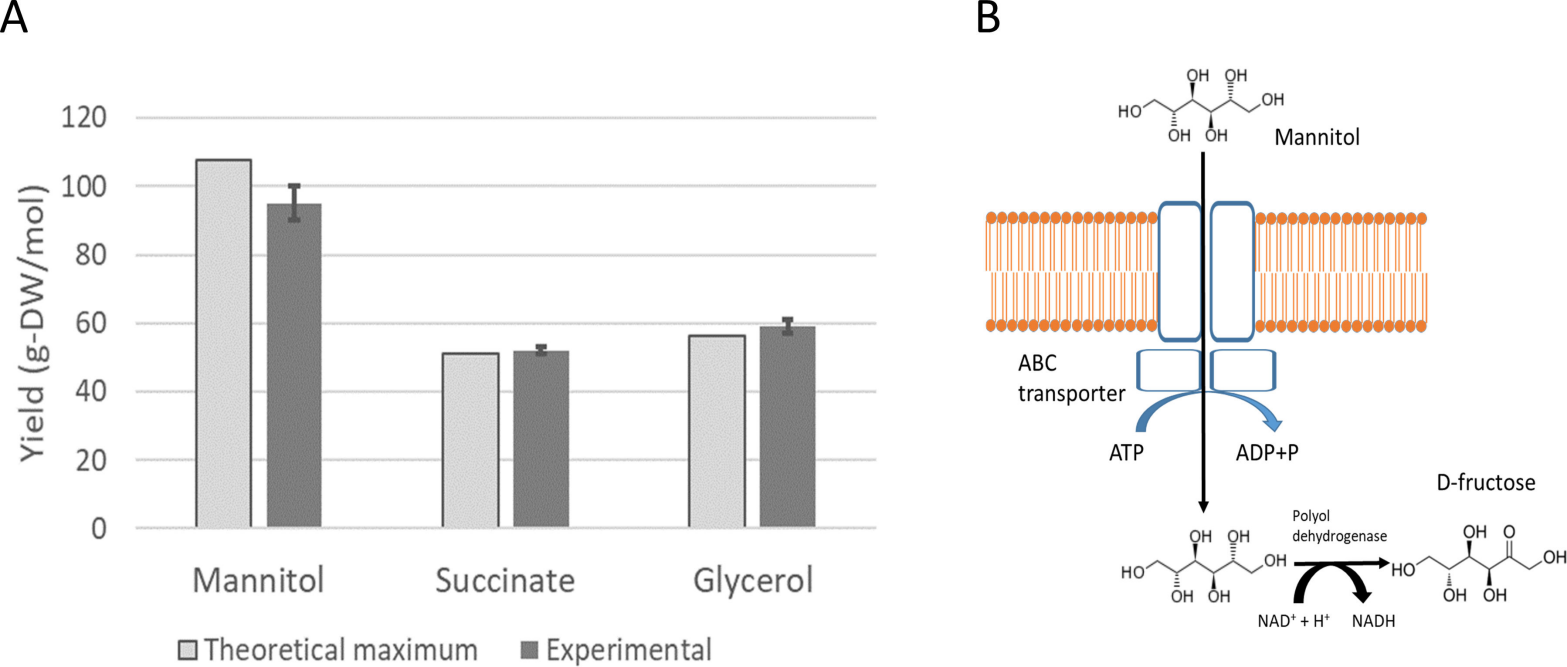
Comparison of experimental biomass yields (with error bars representing standard deviation) on mannitol, succinate, and glycerol and the corresponding theoretical maximum predicted by the model (**A**). Scheme of the initial steps of mannitol metabolism in *P. denitrificans* (**B**).

Experimental maximal yields on mannitol (21), succinate (22), and glycerol (23) have been compared to the model predictions (Fig. 1A). These experimental yields from literature were obtained from experiments in carbon-limited chemostats operated at different dilution rates by calculating the slope of Pirt’s equation. The maximal theoretical yields predicted by FBA were 107.4, 51, and 56.2 g-DW mol^−1^ for mannitol, succinate, and glycerol, respectively. The experimental values reported in literature are 95 ± 5, 52 ± 1, and 59 ± 2 g-DW mol^−1^, with the error intervals showing standard deviations.

The genome annotation did not reveal the presence of a mannitol phosphotransferase system, which transfers phosphate to mannitol from phospho-L-histidine (24), allowing the resulting mannitol-1-phosphate to be further transformed into glucose-6-phosphate. An alternative for mannitol utilization is its oxidation to D-fructose by mannitol dehydrogenase (EC 1.1.1.67). The annotated genome contains a gene annotated as a transcriptional regulator of mannitol utilization (peg.4330 in the RAST annotation) located close to a generic polyol-specific dehydrogenase (peg.4333) and the four subunits of a polyol-specific ABC transporter (peg.4334–peg.4337). Thus, mannitol uptake was assumed to be associated to the consumption of one ATP molecule followed by its oxidation to D-fructose yielding NADH.

### Metabolism of C1 compounds

*Paracoccus denitrificans* uses methanol and formate as single carbon and energy sources (25). These substrates are fully oxidized to CO_2_, which is fixed via the Calvin cycle (26). The genome of *P. denitrificans* contains a gene annotated as serine hydroxymethyl transferase (peg.946 in our RAST annotation), which is involved in the assimilation of C1 compounds in type II methanotrophs. However, the previously mentioned enzyme appears to be inactive, and C1 compounds are assimilated using the Calvin cycle (26). *P. denitrificans* has a large gene cluster, formed by 11 genes, coding methanol dehydrogenase (EC 1.1.2.7) and some of its auxiliary proteins (peg.3083–peg.3093 in our RAST annotation). Methanol dehydrogenase uses pyrroloquinoline quinone (PQQ) as intermediate electron acceptor, which transfers electrons to cytochrome-c with the extrusion of two protons. Methanol oxidation takes place in the periplasmic space. Formaldehyde is further transported into the cytosol. The existence of a formaldehyde-specific transporter has been hypothesized (27), but the corresponding protein has not been identified. Formaldehyde is oxidized to formic acid in the cytosol by a system involving three enzymes: glutathione-dependent formaldehyde-activating enzyme (EC 4.4.1.22; peg.15), S-hydroxymethyl-glutathione dehydrogenase (EC 1.1.1.284; peg.16), and S-formylglutathione hydrolase (EC 3.1.2.12; peg.19) (28). The genome annotation also contains a glutathione-independent NAD-specific formaldehyde dehydrogenase (EC 1.2.1.46) coded by the gene peg.1225. However, mutations in S-hydroxymethyl-glutathione dehydrogenase have been observed to result in loss of the capacity to grow on methanol (29), which means that the gene peg.1225 cannot compensate the loss of this catalytic function. *P. denitrificans* contains an NAD-dependent formate dehydrogenase composed of three subunits (peg.2939, peg.2940, and peg.2941) and a formate dehydrogenase O, composed of four subunits (peg.2910, peg.2911, peg.2912, and peg.2913), which uses membrane soluble quinones as electron acceptors (30). The use of different electron acceptors will lead to different biomass yields on C1 compounds.

**Fig 2:**
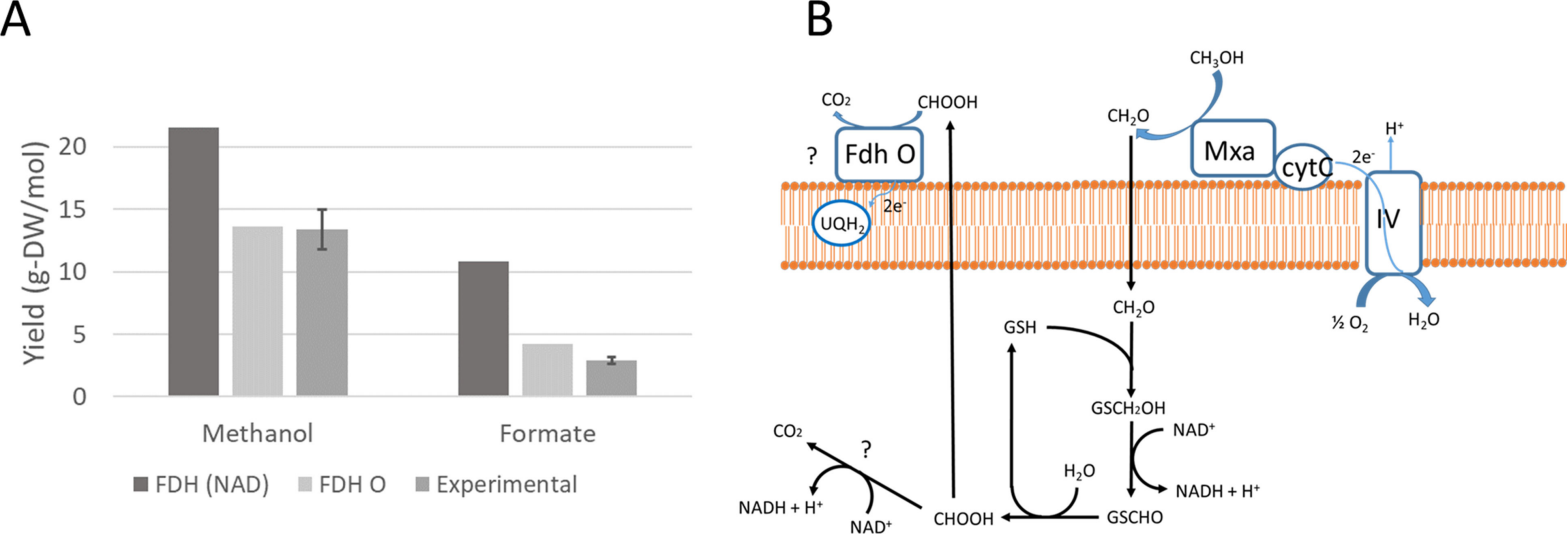
Comparison of the model yield predictions assuming each of the two possible mechanisms of formate oxidation and experimental yields (**A**). Schema of the possible mechanisms of utilization of C1 compounds (**B**).

The reconstructed GSMM has been used to predict maximum theoretical yields on methanol and formate, considering both scenarios. The results have been compared to experimental biomass yields obtained in chemostat cultures (25). In the case of methanol, the observed yield was 13.4 ± 1.6 g-DW mol^−1^, while the maximum theoretical yields were 21.5 g-DW mol^−1^ for NAD-dependent formate dehydrogenase and 13.6 g-DW mol^−1^ for formate dehydrogenase O. In the case of formate, the experimental biomass yield was 2.9 ± 0.3 g-DW mol^−1^, while the maximum theoretical yields were 10.8 g-DW mol^−1^ for NAD-dependent formate dehydrogenase and 4.2 g-DW mol^−1^ for formate dehydrogenase O. The theoretical yields calculated, assuming an NAD-dependent dehydrogenase, clearly overestimate the experimental values, while the hypothesis of formate oxidation by formate dehydrogenase O is consistent with the experimental biomass yields. A scheme of methanol metabolism is depicted in Fig. 2.

### Regulation of ribulose biphosphate carboxylase in order to maximize biomass yield on two carbon sources

*P. denitrificans* co-consumes mannitol and methanol (21). Mannitol and methanol consumption rates were obtained from chemostat experiments fed with different methanol/mannitol concentration ratios (21). The parameters of Pirt’s equation describing mannitol and methanol uptake rates were adjusted for each of the tested concentration ratios (21). Ten different concentration ratios, including pure mannitol and pure methanol, were tested. The main observation by van Verseveld and co-workers (21) was that enzymatic activity of ribulose biphosphate carboxylase (RuBisCO) in cell extracts is observed only for methanol/mannitol concentration ratios higher than 2.11. This means that CO_2_ resulting from methanol oxidation starts being assimilated via the Calvin cycle only over the mentioned threshold. For lower methanol/mannitol ratios, the consumed methanol is used as an energy source only, and no carbon from it is assimilated into the biomass. The reconstructed GSMM was used to assess if this activation of RuBisCO leads to the optimization of biomass yield.

GSMMs do not use substrate concentrations as inputs but as metabolic fluxes. From the parameters of Pirt’s equation reported by van Verseveld and co-workers (21), uptake ratios were calculated as a function of the specific growth rate for each concentration ratio. The consumption ratios of mannitol and methanol tend asymptotically to the concentration ratios, and for specific growth rates of 0.3 h^−1^ or higher, both values were equal. Thus, FBA simulations were carried out at 0.3 h^−1^.

Non-growth-associated ATP costs were calculated from the maintenance substrate uptake rates reported by van Verseveld and co-workers (21) (the ordinate at the origin in Pirt’s equation). The GSMM predicts theoretical ATP production yields of 4.9 molATP/molMethanol and 29.7 molATP/molMannitol. This means that the maintenance ATP consumption rate can be obtained as follows:


(2) $$m_{ATP}=4.9m_{methanol}+29.1m_{mannitol}$$

This value was considered to be independent of the methanol/mannitol concentration ratio and was estimated by averaging the results for each of the 10 tested concentration ratios, leading to an estimation of 7.5 ± 2 mmol ATP g-DW^−1^ h^−1^ (the error interval is the standard deviation).

**Fig 3:**
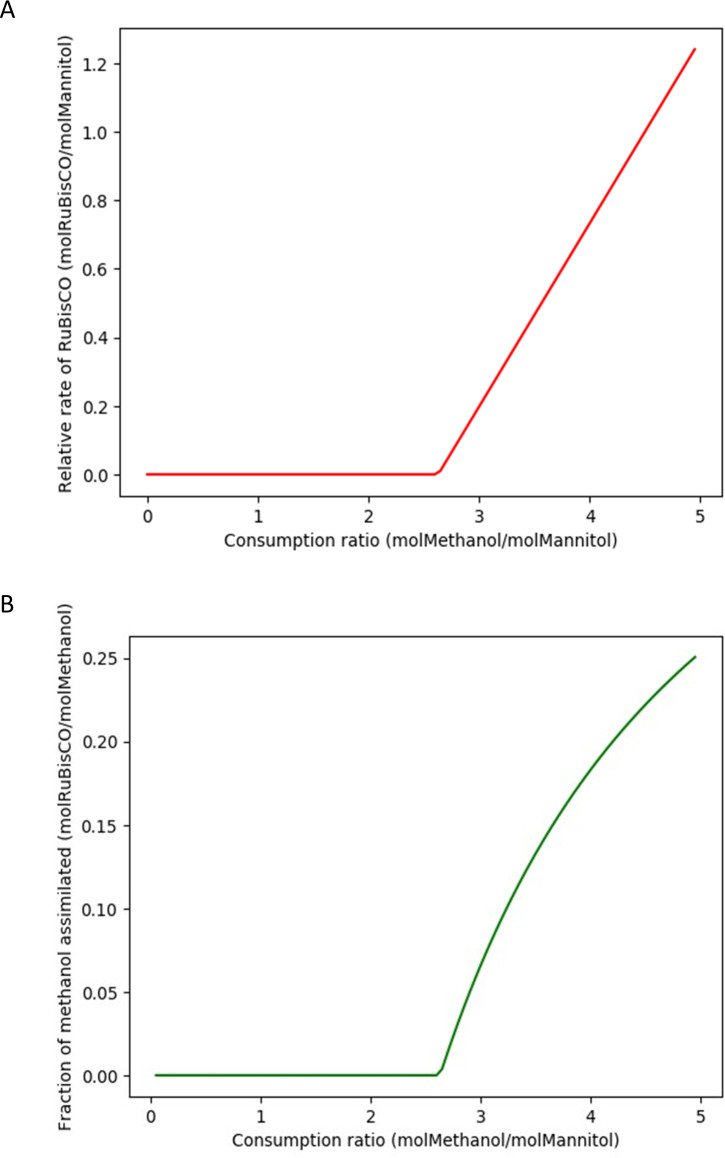
Reaction rate of RuBisCO per mol of consumed mannitol, as a function of the methanol/mannitol uptake ratio (**A**). Fraction of the methanol which is assimilated into biomass versus the methanol/mannitol uptake ratio (**B**).

The metabolic flux of RuBisCO was calculated for different methanol/mannitol uptake ratios as follows: specific growth rate was set to 0.3 h^−1^ and ATP maintenance rate was set to 7.5 mmolATP g-DW^−1^ h^−1^. With the mentioned constraints, a reaction supplying a mixture of methanol and mannitol at a fixed ratio was defined, and its flux was minimized using FBA. This simulation was repeated iteratively with methanol/mannitol ratios ranging from 0 to 5. RuBisCO starts carrying a non-zero flux at flux ratios higher than 2.6 (Fig. 3), which corresponds closely to the observed enzymatic activity in cell extracts (21). From these results it can be concluded that the expression of RuBisCO is tightly regulated in order to optimize biomass production when growing on mixtures of methanol and mannitol.

### Denitrification

#### Growth under anoxic conditions

As it is already indicated by its name, *P. denitrificans* grows in the absence of oxygen using nitrate as final electron acceptor. Thus, the model has to be capable of reproducing such behavior. For anoxic growth to be possible, it is not enough to achieve ATP synthesis through denitrification, but also all the constitutive components of the biomass should be synthesized in the absence of oxygen. One essential biomass component requiring oxygen for its biosynthesis is ubiquinone, which plays a key role as redox carrier in the respiratory chain. A molecule that plays the same role as ubiquinone as redox carrier, but which does not require oxygen for its biosynthesis, is menaquinone. For a long time, menaquinone has been considered as the anaerobic substitute of ubiquinone, and anoxic organisms have been thought to rely on menaquinone (31); however, the menaquinone synthetic pathway appears to be absent from the genome of *P. denitrificans*. Oxygen-independent ubiquinone synthesis has been recently characterized in *E. coli* (32). In absence of oxygen, the three reaction steps that are normally catalyzed by the oxygen-dependent hydroxylases, UbiI, UbiH, and UbiF, are catalyzed instead by an enzymatic complex formed by the proteins, UbiU and UbiV, which are coded in an operon that includes a third gene (UbiT) with regulatory functions. *P. denitrificans* has three orthologous genes organized in an operon with the same arrangement as the operon found in *E. coli* (peg.4386, peg.4387, and peg.4388). This operon has been shown to be essential for denitrification in the bacterium *Pseudomonas aeruginosa* (33), which suggests that it could play the same role in *P. denitrificans*. The electron acceptor involved in the reactions catalyzed by the UbiV-UbiU complex is unknown. The discoverers of this new ubiquinone synthesis mechanism found that both UbiV and UbiU have Fe_4_-S_4_ clusters, similar to those involved in complex II of the respiratory chain (32). Thus, for modeling purposes, we assumed that the final electron acceptor of UbiV-UbiU is ubiquinone (as it is the case in complex II of the respiratory chain).

#### Biomass yields on acetate, formate, and succinate under denitrification conditions

*P. denitrificans* is a model denitrifying organism. This work will assess the predictions of biomass yields on acetate and formate using NO_3_^−^ as the final electron acceptor. Experimental specific growth rates and biomass yields under carbon-limiting conditions using acetate and formate as carbon sources were taken from the literature (34). The specific growth rate and biomass yield on succinate were measured in this work. In the three cases, the biomass yields were obtained from batch experiments, with specific growth rates of 0.14 h^−1^ for acetate, 0.07 h^−1^ for formate, and 0.22 h^−1^ for succinate. As shown in Table 1, the experimental biomass yields were 14.3 ± 2 g-DW mol^−1^ for acetate, 2.64 ± 0.3 g-DW mol^−1^ for formate, and 32.2 ± 1 g-DW mol^−1^ for succinate.

**TABLE 1:** Biomass yields with nitrate as electron acceptor

Electron donor	Experimental biomass yield (g-DW/mol)	Estimated biomass yield (g-DW/mol)
Acetate	14.3 ± 2	14.7
Formate	2.6 ± 0.3	1.8
Succinate	32 ± 1	33.9

Theoretical yields were calculated using FBA by setting the oxygen consumption to zero, allowing uptake of nitrate and setting the specific growth rate to its experimental value. The ATP maintenance consumption was previously found to be in the interval 7.5 ± 2 mmol ATP g-DW^−1^ h^−1^. Using the lowest value of the mentioned interval led to better yield predictions in all the subsequent simulations of batch growth presented in this article, thus the ATP maintenance consumption was set to 5.5 mmol ATP g-DW^−1^ h^−1^, and the uptake rate of the carbon source was minimized. The predicted yields were 15.2 ± 1 g-DW mol^−1^ for acetate, 1.8 ± 0.1 g-DW mol^−1^ for formate, and 36 ± 1 g-DW mol^−1^ for succinate (Table 1).

Acetate utilization was modeled considering acetyl-CoA synthesis with the transformation of an ATP molecule into AMP. This enzymatic activity corresponds to acetyl-CoA synthase (EC 6.2.1.1) and was already previously reported in the literature (34); the gene responsible for this enzymatic activity is peg.983, according to the RAST annotation.

### Growth on non-conventional substrates

*P. denitrificans* is a soil organism with the capability of catabolizing several non-conventional substrates. During the process of model curation, pathways involved in the catabolism of adipic acid, 1,4-butanediol, 1,3-propanediol, and ethylene glycol were identified. The growth on these substrates was experimentally verified, and the model predicted accurately the biomass yields in all these substrates.

#### Metabolism of adipic acid

The genome of *P. denitrificans* has two genes (peg.4995 and peg.5206) annotated as 3-oxoadipyl-CoA thiolase (Ec 2.3.1. 174), which breaks down 3-oxoadipyl-CoA into acetyl-CoA and succinyl-CoA. This is the last step of the catabolism of adipic acid. There are 7 different genes annotated as putative 3-hydroxyacyl-CoA dehydrogenases (EC 1.1.1.35) and 12 putative enoyl-CoA hydratases (EC 4.2.1.17). Three of these genes have been annotated by RAST as having both functions simultaneously (peg.203, peg.2954, and peg.3321). Thus, *P. denitrificans* is likely to metabolize adipic acid. In order to confirm the presence of this pathway, BLAST was used to identify orthologs of the dca genes in *Acinetobacter* sp. ADP1 (35), which in *Acinetobacter* forms two operons oriented in opposite senses that share the same regulatory region. The genes dcaJ and dcaI of *Acinetobacter* sp. ADP1 (adipate CoA transferases) showed high homology to peg.5205 and peg.5204. These two genes form an operon with peg.5206 (3-oxoadipyl-CoA thiolase). Thus, the initial and final steps of the adipic acid degradation pathway seem to be under common regulation. The three intermediate steps, in *Acinetobacter,* are carried out by the genes dcaA, dcaE, and dcaH, which had three orthologs clustered in the same region while not forming a single operon (peg.199, peg.200, and peg.203).

The BLAST results are summarized in Table 2; the growth of *P. denitrificans* in adipic acid was confirmed experimentally, and the observed biomass yield was consistent with the model prediction (as it is shown in the following section).

**TABLE 2:** BLAST scores for the enzymes involved in the metabolism of adipic acid

Protein from *Acinetobacter* sp. ADP1	BLAST matches in the proteome of *P. denitrificans*	*E*-scores from BLAST
dcaJ	peg.5205	*E* = 4 × 10^−78^
dcaI	peg.5204	*E* = 9 × 10^−74^
dcaA	peg.200	*E* = 0
dcaE	peg.203	*E* = 5 × 10^−22^
dcaH	peg.199	*E* = 3 × 10^−100^
dcaF	peg.5206	*E* = 2 × 10^−82^

#### Metabolism of 1,4-butanediol

The genome of *P. denitrificans* contains three different putative succinate semialdehyde dehydrogenases (EC 1.2.1.16), which catalyze the oxidation of succinate semialdehyde to succinic acid (peg.246, peg.2316, and peg.5151). This reaction was absent from the draft generated by SEED and had to be added manually. Oxidation of succinate semialdehyde is the last step of the 1,4-butanediol assimilation pathway. This compound is one of the constitutive monomers of polyesters, such as PBS, PBSA, PBST, PBSTIL, and PBAT, and has also toxic effects for humans. 1,4-Butanediol is used as carbon and energy source by *Pseudomonas putida* KT2440, and the dehydrogenases involved in its metabolism have been identified recently (36). The native enzymes of *P. putida* KT2440 catalyzing the first three oxidation steps of 1,4-butanediol metabolism were compared by BLAST to the proteome of *P. denitrificans*. The BLAST output file is provided as supplementary material, and the results are summarized in Table 3.

**TABLE 3:** BLAST scores for the dehydrogenases involved in the metabolism of 1,4-butanediol

Protein from *P. putida* KT2440	BLAST matches in the proteome of *P. denitrificans*	*E*-scores from BLAST
PP_2674	peg.20 peg.3083	*E* = 4 × 10^−89^ *E* = 2 × 10^−86^
PP_2680	peg.2425 peg.5153	*E* = 0 *E* = 10^−80^
PP_2049	peg.245 peg.4723	*E* = 10^−63^ *E* = 2 × 10^−54^

**Fig 4:**
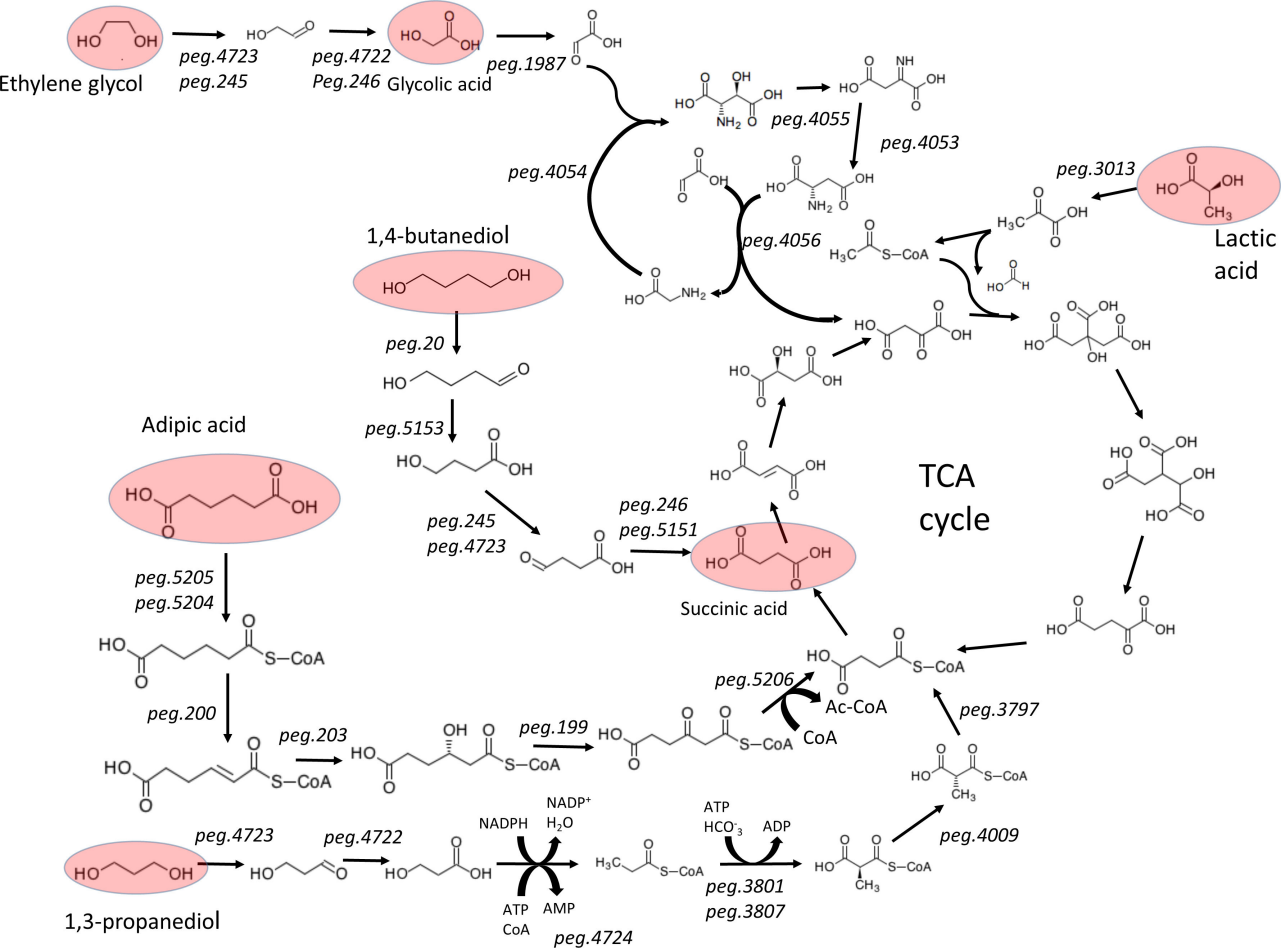
Biodegradation pathways of non-conventional substrates in *P. denitrificans*. The metabolites marked in red have been used as substrates in the batch fermentations discussed in the next section.

The BLAST results (Table 2) revealed that the first oxidation step of 1,4-butanediol is likely to be catalyzed by two genes annotated as methanol dehydrogenases (peg.20 and peg.3083), which use PQQ as intermediate electron acceptor, and transfers electrons to cytochrome-c with the extrusion of two protons. This reaction takes place in the periplasm. In the absence of evidence for a specific transporter, the transfer of the resulting 4-hydroxybutyraldehyde was assumed to occur without ATP consumption. Of the two best BLAST hits for the 4-hydroxybutyraldehyde dehydrogenase (PP_2680), one of them (peg.5153) is placed in the same operon as succinate semialdehyde dehydrogenase (peg.5151), which suggests a functional association between both genes. The gene peg.5151, situated between them in the same operon, is also a metabolic gene-coding malate dehydrogenase (EC 1.1.1.83). This enzyme is involved in the assimilation of succinate (or any succinate precursor such as 1,4-butanediol) by producing pyruvate, which is then transformed into acetyl-CoA, which feeds the TCA cycle. Among the best BLAST hits for 4-hydroxybutyrate dehydrogenase (PP_2049), we find the gene peg.245, which forms an operon with the succinate-semialdehyde dehydrogenase peg.246, suggesting again a functional relationship between both the genes. The postulated metabolic pathway for 1,4-butanediol via succinate is summarized in Fig. 4A.

*P. denitrificans* was found experimentally to grow on 1,4-butanediol with a specific growth rate of 0.022 ± 0.001 h^−1^. This value and the previously estimated ATP maintenance costs of 7.5 ± 2 mmol ATP g-DW^−1^ h^−1^ were used to estimate biomass yield on 1,4-butanediol. The estimated yield was 42 ± 6 g-DW mol^−1^ (the error interval was calculated from the error in ATP maintenance), which compares satisfactorily with the experimental biomass yield of 42 ± 2 g-DW mol^−1^ (the error corresponds to the standard deviation of three fermentations).

#### Metabolism of ethylene glycol

Ethylene glycol, a by-product of many industrial processes, is not metabolized by wild-type *E. coli,* but it is utilized by mutant strains such as *E. coli* K-12, which expresses at high level the genes: glycolaldehyde reductase (fucO) and glycolaldehyde dehydrogenase (aldA). In *E. coli*, these two genes are located in two completely different operons (37). BLAST was used to identify genes with the same putative function in *P. dentitrificans*. Among the most similar candidates, two pairs of genes are organized forming operons in which both genes overlap in four nucleotides. The BLAST scores for these genes are reported in Table 4.

**TABLE 4:** BLAST scores for the genes fucO and aldA, involved in the oxidation of ethylene glycol

Protein from *E. coli* K-12	BLAST matches in the proteome of *P. denitrificans*	*E*-scores from BLAST
fucO	peg.4723 peg.245	*E* = 7 × 10^−51^ *E* = 10^−42^
aldA	peg.4722 peg.246	*E* = 10^−51^ *E* = 8 × 10^−89^

The next step in the degradation of ethylene glycol is the oxidation of glycolic acid to glyoxylate, which is catalyzed by the enzyme glyoxylate reductase (coded by the gene peg.1987 in our annotation).

The glyoxylate resulting from the oxidation of glycolic acid is further metabolized via the β-hydroxyaspartate cycle, a pathway recently discovered and characterized by Schada von Borzyskowski and co-workers (38). This cycle is composed of four reactions whose enzymes are coded in a single operon in *P. denitrificans* (peg.4053–peg.4056 in our RAST annotation).

*P. denitrificans* was shown experimentally to grow on ethylene glycol, and the observed biomass yield was consistent with the prediction of the model (as it is shown in Table 6).

#### Metabolism of 1,3-propanediol

Given the fact that *P. denitrificans* grows on both ethylene glycol and 1,4-butanediol, we aimed to test if it also grows on 1,3-propanediol. BLAST was used to identify an oxidation pathway of 1,3-propanediol. The following sequences were used as queries: 1,3-propanediol dehydrogenase from *Clostridium pasteurianum* (39), with GenBank accession number AF006034.2; 3-hydroxypropionaldehyde dehydrogenase from *E. coli* K-12 (40), with Uniprot accession number P23883; and propionyl-CoA synthase from *Chloroflexus aurantiacus* (41), with Uniprot accession number A9WEU4. The analysis (Table 5) revealed three genes forming an operon in *P. denitrificans*, which are responsible for the mentioned biodegradation pathway (peg.4723, peg.4722, and peg.4724). The BLAST results are presented in Table 5.

**TABLE 5:** BLAST scores for the enzymes involved in the metabolism of 1,3-propanediol

Enzyme from the literature	BLAST matches in the proteome of *P. denitrificans*	*E*-scores from BLAST
AF006034.2	peg.4723	*E* = 6 × 10^−66^
A9WEU4	peg.4724	*E* = 3 × 10^−62^
P23883	peg.4722	*E* = 3 × 10^−46^

Propionyl-CoA is further metabolized by *P. denitrificans* using two alternative pathways: the methylmalonyl-CoA pathway (MMCP) and the methylcytrate cycle (MCC) (42). MMCP starts with an ATP consuming carboxylation step, catalyzed by a complex of two proteins coded by peg.3801 and peg.3807 (in our RAST annotation). MCC starts with the condensation of propionyl-CoA with oxaloacetate, which yields 2-methylcitrate. Both pathways are present in the model and can be used alternatively resulting in identical biomass yields.

It was shown experimentally that *P. denitrificans* grows on 1,3-propanediol as sole carbon and energy source, and the observed biomass yield was consistent with the predictions of the model (Table 6). The predicted yield is the same using both MCC and MMCP for the metabolism of propionyl-CoA.

**TABLE 6:** Experimental growth rates in different substrates and predicted versus experimental biomass yields^*a*^

	*µ* (h^−1^)	*Y* (g.mol^−1^) Experimental	*Y* (g.mol^−1^) Theoretical
Succinic acid	0.232 ± 0.002	48.4 ± 2.1	47.3
Lactic acid	0.215 ± 0.008	42.9 ± 1.8	42.5
Glycolic acid	0.167 ± 0.003	16.1 ± 0.6	20.8
1,3-Propanediol	0.049 ± 0.001	40.1 ± 2	39.3
Ethylene glycol	0.045 ± 0.001	26.9 ± 0.1	27.8
Adipic acid	0.037 ± 0.001	68.1 ± 6.8	65.1
1,4-Butanediol	0.022 ± 0.001	44.4 ± 0.5	45.6

^*a*^ The error bars correspond to standard deviations (*n* = 3).

### Prediction of yields in batch fermentations

In order to test the performance of the model, *P. denitrificans* was cultured in the presence of oxygen under batch conditions, using seven different substrates as single carbon and energy sources . Those substrates are the previously mentioned non-conventional substrates, glycolic acid, which is an intermediate of the degradation of ethylene glycol, succinic acid (the most common substrate for this bacterium), and lactic acid, a common substrate that, nevertheless, is not used by some model organisms such as *Bacillus subtilis*.

Growth in batch was simulated by setting the ATP maintenance consumption rate to a value of 5.5 mmolATP g-DW^−1^ h^−1^. The growth rate was fixed to its experimental value. The uptake rate of the carbon source was minimized. As it can be seen in Table 6, the model accurately predicted the biomass yields for each of the seven substrates.

## DISCUSSION

In this study, we present the first functional genome-scale metabolic model of *Paracoccus denitrificans*, a broadly studied organism with high metabolic versatility. An automatically reconstructed model of *P. denitrificans* has been previously published: https://github.com/cdanielmachado/embl_gems/tree/master/models/p/paracoccus. However, this model does not predict growth in any single carbon source (among other artifacts, it requires the uptake of AMP to grow in any carbon source). This shows that an accurate manual curation of automatically generated drafts is still necessary in order to obtain functional models that can be used to predict realistic metabolic flux distributions and outputs of genetic manipulations.

The model was tested by comparing its ability to predict biomass yields on different carbon sources and electron acceptors. The reconstructed GSMM was able to predict the fact that, when grown on a mixture of mannitol and methanol, the enzyme RuBisCO, responsible for the assimilation of C1 compounds via the Calvin cycle, is only expressed over a certain threshold of methanol/mannitol consumption ratio, leading to the maximization of biomass yield. The capacity of *P. denitrificans* to grow on non-conventional carbon sources, such as adipic acid, 1,4-butanediol, ethylene glycol, and 1,3-propanediol, was predicted based on the model annotation and was further confirmed experimentally. The criteria of optimization of the biomass yield led to accurate predictions for all the 10 different substrates tested. The modeling of denitrification was also accurate.

The metabolic network of *P. denitrificans* shows functional degeneracy in the utilization of C3 and C2 carbon sources. As it has been discussed in the Results section, propionyl-CoA can be metabolized by two alternative pathways (MMCP and MCC), and both of these pathways result in the same theoretical biomass yield. In the same way, two metabolic pathways for the utilization of acetyl-CoA when growing on C2 substrates are present in *P. denitrificans* (42). These pathways are the widespread glyoxylate cycle (GC) and the much rarer ethylmalonyl-CoA pathway (EMCP). Both pathways lead to the same theoretical biomass yield on C2 compounds. The yield in acetate matches the experimental yield (Table 1). A recent article (41) has reported that the presence of intermediates of the EMCP triggers the expression of enzymes involved in the GC; thus, a regulatory reason may explain this functional redundancy in the genome of *P. denitrificans*.

This fist functional GSMM of *P. denitrificans* can be used as a scaffold for the design of metabolic engineering strategies, enabling the use of *P. denitrificans* as a cell factory for industrial biotechnology.

## MATERIALS AND METHODS

### Model reconstruction and FBA simulation

The genome of *Paraccocus denitrificans* was obtained from GenBank (GCA_000203895.1). After annotation with RAST (17), a draft metabolic reconstruction was generated with SEED (18). The draft was manually curated as described elsewhere (43), by checking the gene annotations of each reaction and cross-checking the RAST annotation of each metabolic gene with the KEGG entry of each putative enzymatic activity. Model manipulations and FBA simulations were carried out using the python library COBRApy (44), version 0.26.2 with python 3.10.6. The model in SBML format (Paracoccus_denitrificans.xml), the MEMOTE report, and the associated lists of metabolites (Paracoccus_metabolites.txt) and reactions (Paracoccus_reactions.txt) in tab separated formats have been deposited in https://github.com/SergioBordel/ModelParacoccus. The RAST annotation, both in Excel and GenBank formats, has been placed in the same repository.

### Culture of *P. denitrificans*

*P. denitrificans* [Pd1222 Rif^R^, (12)] was cultivated in 200 mL batch cultures at 37°C with constant shaking (200 rpm). Precultures of *P. denitrificans* were grown aerobically in LB with 50 µg·mL^−1^ of rifampicin. Cultures were performed in mineral salt medium MSM (adjusted to pH = 7) containing phosphate and nitrogen, with the following composition (g/L): KH_2_PO_4_ (0.95), K_2_HPO_4_ (2.27), (NH_4_)_2_SO_4_ (0.67), trace elements solution and each of the plastic monomers as the sole carbon source (3 g/L), i.e., 25.4 mM of succinic acid, 26.7 mM of lactic acid, 20.5 mM of adipic acid, 33.3 mM of 1,4-butanediol, 39.4 mM of 1,3-propanediol, 48.3 mM of 1,2-etanediol, and 39.4 mM of glycolic acid.

### Culture of *P. denitrificans* under anoxic conditions

*P. denitrificans* anoxic cultures were conducted in 120 mL serum bottles (hermetically closed with an isoprene rubber and an aluminum crimp seal) containing 100 mL of MSM (with 25 mM succinate as sole carbon source). The medium composition was changed as follows: the nitrogen source (NH_4_)_2_SO_4_ was substituted with KNO_3_ (50 mM) and Na_2_SO_4_ (5 mM). After inoculation of the medium with 2.5 mL of the preculture, the oxygen was removed from the bottles by gasification with helium. Cultures were incubated for several days at 37°C under orbital shaking at 200 rpm and sampled equally than the aerobically cultures.

### Analytical methods

#### Quantification of biomass

Biomass of *P. denitrificans* was measured spectrophotometrically using a SPECTROstar Nano spectrophotometer (BMG Labtech). OD was measured at 600 nm. OD was correlated with dry weight of *P. denitrificans*. For dry weight determination, three culture samples (2 mL) were washed twice with MilliQ water and dried for 5 days at 80°C. The OD-to-biomass relation obtained is biomass (g·L^−1^) = OD600 nm × 0.37 g·L^−1^. For OD values higher than 1, the sample was diluted, in order to be within the linear range of the spectrophotometer.

#### 1,4-Butanediol, 1,3-propanediol, 1,2-etanediol, and adipic acid quantification

Samples were collected from 2 mL culture samples after centrifugation of the samples (14,000 rpm for 5 min at 4°C) and filtering of the supernatant using Nylon syringe filters of 0.22 µm. Samples were then analyzed using an Agilent 7820A GC coupled with a 5977E MSD (Agilent technologies, Santa Clara, USA) equipped with a DB-wax column (30 m × 250 μm × 0.25 μm). The detector and injector temperatures were kept constant at 250°C, and the oven temperature was increased from 50°C to 220°C at 10°C min^−1^ and maintained at 220°C for 2  min, before being increased again at 5°C min^−1^ until reaching 240°C. Instrument linearity was evaluated with 1,4-butanediol, 1,3-propanediol, 1,2-etanediol, and adipic acid (Sigma-Aldrich) in the concentration range of 2–60 mM.

#### Succinic acid, lactic acid, and glycolic acid quantification

Samples were collected from 2 mL culture samples after centrifugation of the samples (14,000 rpm for 5  min at 4°C) and filtering of the supernatant using Nylon syringe filters of 0.22 µm. Samples were measured by HPLC through an Alliance Waters HPLC equipped with an Aminex HPX-87H column (7.8 mm × 300 mm). The mobile phase was H_2_SO_4_ (25 mM), the column temperature was 75°C, and the eluent flow rate was 0.7 mL min^−1^. Instrument linearity was evaluated with each of the compounds (obtained from Sigma-Aldrich; ReagentPlus, 99%) in the concentration range of 0.8–90 mM.

## Data Availability

The genome scale metabolic model can be downloaded at: https://github.com/SergioBordel/ModelParacoccus
